# Novel Exon of Mammalian ADAR2 Extends Open Reading Frame

**DOI:** 10.1371/journal.pone.0004225

**Published:** 2009-01-19

**Authors:** Stefan Maas, Willemijn M. Gommans

**Affiliations:** Department of Biological Sciences, Lehigh University, Bethlehem, Pennsylvania, United States of America; Universität Heidelberg, Germany

## Abstract

**Background:**

The post-transcriptional processing of pre-mRNAs by RNA editing contributes significantly to the complexity of the mammalian transcriptome. RNA editing by site-selective A-to-I modification also regulates protein function through recoding of genomically specified sequences. The adenosine deaminase ADAR2 is the main enzyme responsible for recoding editing and loss of ADAR2 function in mice leads to a phenotype of epilepsy and premature death. Although A-to-I RNA editing is known to be subject to developmental and cell-type specific regulation, there is little knowledge regarding the mechanisms that regulate RNA editing in vivo. Therefore, the characterization of ADAR expression and identification of alternative ADAR variants is an important prerequisite for understanding the mechanisms for regulation of RNA editing and the causes for deregulation in disease.

**Methodology/Principal Findings:**

Here we present evidence for a new ADAR2 splice variant that extends the open reading frame of ADAR2 by 49 amino acids through the utilization of an exon located 18 kilobases upstream of the previously annotated first coding exon and driven by a candidate alternative promoter. Interestingly, the 49 amino acid extension harbors a sequence motif that is closely related to the R-domain of ADAR3 where it has been shown to function as a basic, single-stranded RNA binding domain. Quantitative expression analysis shows that expression of the novel ADAR2 splice variant is tissue specific being highest in the cerebellum.

**Conclusions/Significance:**

The strong sequence conservation of the ADAR2 R-domain between human, mouse and rat ADAR2 genes suggests a conserved function for this isoform of the RNA editing enzyme.

## Introduction

Nuclear pre-mRNA editing has been recognized as an important mechanism for the generation of protein diversity in mammals (for review see [1], [2]). In A-to-I RNA editing, specific adenosine bases in pre-mRNAs undergo deamination to inosine, which is interpreted as guanosine by the translation machinery and therefore can lead to single amino acid substitutions in protein products that result from edited mRNAs.

A few dozen genes are known in human that undergo RNA editing in their pre-mRNA at specific locations resulting in protein product variants with altered functions (for review see [3]). In the case of the glutamate receptor subunit GluR-2 the single amino acid substitution (Q-to-R) induced by editing is critical for normal brain function [4]. In addition to these so-called ‘recoding’ editing events, many pre-mRNAs undergo A-to-I modification in untranslated exonic, and in non-coding intronic sequences. These include the widespread editing of Alu-type repeat elements in the human transcriptome [5]–[7] and the editing of micro RNA precursors [8]–[10]. The deficiency or hyperactivity of A-to-I RNA editing has been linked to human disease phenotypes, such as epilepsy, malignant brain cancer, amyotrophic lateral sclerosis, immunological disorders and depression (for review see [11]).

It is not completely understood what determines which adenosine in a pre-mRNA molecule will be targeted for deamination in vivo, but essential prerequisites are secondary structures in the RNA substrate that include double-stranded components within the vicinity of the editing site on one hand, and the presence of an adenosine deaminase on the other. In mammals, two adenosine deaminases that act on RNA (ADARs) have been characterized, which mediate all of the currently known A-to-I editing events [1]. The two enzymes harbor double-stranded RNA binding domains as well as a catalytic deaminase domain that is evolutionary related to tRNA specific adenosine deaminases, and more distantly to cytidine deaminases. A third enzyme, ADAR3 [12], shares high sequence similarity with ADAR2 and is present in vertebrate species, but to date has not been demonstrated to possess A-to-I RNA editing activity.

ADAR1 and ADAR2 generally show overlapping activity profiles on a given RNA substrate with some of the known editing sites being targeted predominantly by one of the two enzymes [3]. Both ADAR1 and ADAR2 are subject to alternative splicing in mammals creating protein variants of different lengths, in some cases with altered activity [13]–[17]. ADAR1 is further known to be expressed either by an interferon regulated promoter leading to the production of ADAR1p150, or by one of two downstream promoters that result in the synthesis of ADAR1p110. The two versions of ADAR1 display distinct intracellular distribution and probably fulfill distinct cellular functions [18], [19].

Mammalian ADAR2 is an essential gene due to the fact that it alone is responsible for editing of the GluR-2 Q/R editing site and the loss of that function results in early death due to hyperexcitability of principal neurons [4]. The regulation of RNA editing activity in vivo is still largely unknown. Therefore, the characterization of ADAR2 transcription and alternative processing is an important prerequisite for understanding the intracellular regulation of RNA editing.

The ADAR2 gene has been characterized to encompass 14 exons in human [17] and several alternative splicing events have been identified [13], [15], [16], [20]. For example, one involving inclusion of alternative exon 5a, which introduces a 120 nucleotide coding Alu-repeat sequence in frame, and another where self-editing of the ADAR2 pre-mRNA creates a 3′-prime splice site within intron 1 leading to the inclusion of 47 nt of intronic sequence [13], [15], [16], [20].

In this study we present evidence for a new ADAR2 variant that utilizes a previously undescribed exon that is likely expressed from an alternative promoter. Importantly, the alternative splicing event extends the open reading frame of ADAR2 and is conserved across vertebrates. Interestingly, the 49 amino acid extension is closely related in sequence to the N-terminal region of ADAR3, which has been shown to possess single-stranded RNA binding activity [21]. Although A-to-I RNA editing is known to be subject to developmental and cell-type specific regulation, there is little knowledge regarding the mechanisms that regulate RNA editing in vivo. Therefore, the characterization of ADAR expression and identification of alternative ADAR variants is an important prerequisite for understanding the mechanisms for regulation of RNA editing and the causes for deregulation in disease.

## Results and Discussion

### An alternative ADAR2 spliceform that extends the open reading frame

**Figure 1:**
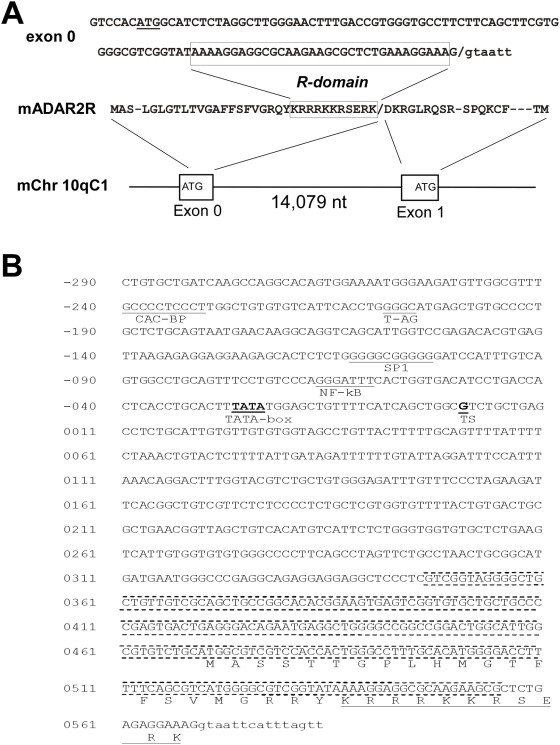
A new translated exon in ADAR2. A) Mouse exon 0 nucleotide, amino acid sequence and details of mAdar2 gene structure. The sequence of the R-domain is boxed, the translational start codon underlined. B) Human ADAR2 exon 0. The exon 0 sequence on human chromosome 21 is shown including translation and promoter sequences and transcriptional start site (TS) as predicted by ProScan. The CpG island is indicated by dotted lines above and below the sequence. Putative transcription factor binding sites for NF-κB, CAC-BP, T-AG, SP1 and a predicted TATA box sequence are marked. Intronic sequences are in small letters.

While characterizing the murine Adar2 gene [4], we performed 5′-RACE experiments and noticed a rare Adar2 cDNA species in mouse brain that extended the open reading frame of the protein N-terminally by 49 amino acids (S. Maas, M. Higuchi and P.H. Seeburg, unpublished observation). We then asked if this sequence is encoded by a previously unrecognized exon in the Adar2 gene. Indeed, within the mouse genome sequence, we were able to locate the corresponding nucleotide sequence on chromosome 10 region qC1, which is followed by a 5′-splice consensus sequence indicating the beginning of the adjacent intron (see Figure 1A).

**Figure 2:**
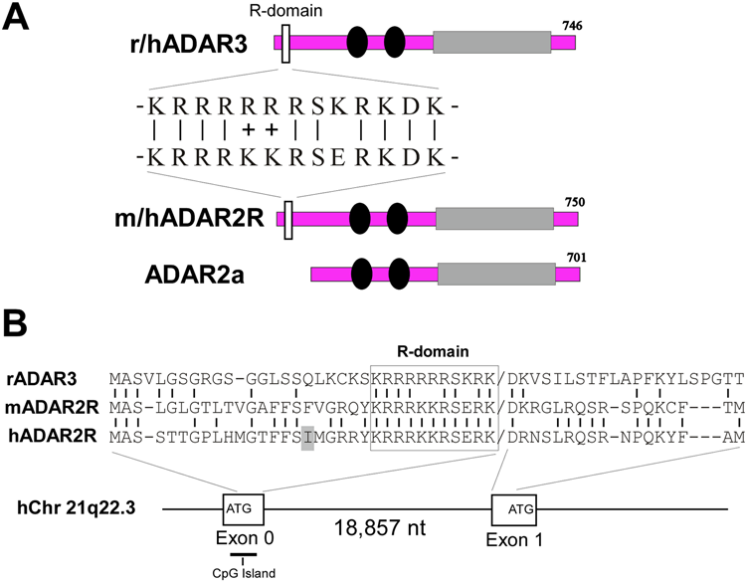
Comparison of ADAR2R with ADAR3 R-domain. A) Comparison of the rat and human ADAR3 protein with the mouse and human ADAR2R protein variant. Includes sequence alignment of ADAR3 R-domain and the putative R-domain of ADAR2R. Black circles: dsRNA binding domain, shaded rectangles: catalytic deaminase domain. B) Alignment of the rat ADAR3 N-terminal region and the translated sequence of the mouse and human ADAR2R N-terminus. The R-domain sequence is boxed and identical amino acids are indicated by a vertical line. Below a schematic representation of the relative position of exon 0 on human Chromosome 21 at the ADAR2 gene locus. The grey-colored Isoleucine residue (I) in hADAR2R represents the position of a recoding SNP that leads to a I-to-V change.

Using the 49 amino acid mouse sequence we searched the human genome sequence using the tblastn protocol (NCBI). We identified a closely related nucleotide sequence within the human ADAR2 gene on chromosome 21. It is located ca.18 kb 5′ of the sequence designated as exon 1 in the human ADAR2 gene [20] and is followed by a consensus 5′ splice donor sequence. Figure 2B shows the alignment of the human and mouse exon 0 sequences and a schematic indicating the relative position of exon 0 within the human ADAR2 gene. In order to produce the novel ADAR2 protein variant, exon 0 becomes spliced to exon 1, which harbors the translational start site for the major ADAR2 splice form. In our 5′-RACE analysis of the mouse Adar2 gene the different 5′-sequences upstream of exon 1 indicated that exon 0 is probably expressed from a different promoter than the one used for the expression of the regular ADAR2 splice variants. There is no discernable consensus upstream splice site for exon 0, and the only mouse EST sequence that includes exon 0 as well as the single cDNA clone we obtained from mouse brain, lack any further upstream sequences derived from additional untranslated exons. The fact that the 5′-region of exon 0 overlaps with the location of a CpG-Island (according to UCSC human genome browser), further supports the notion that a transcriptional start site is close by. Generally, CpG islands are associated with genes, particularly housekeeping genes, in vertebrates. CpG islands are particularly common near transcripton start sites, and are often associated with promoter regions. In this case, the CpG island is 209 bp long with a GC content of 63.3%. It spans from chromosome nt 43077313 to 43077521 (see Figure 1B).

Indeed, when we apply the algorithm ProScan (version 1.7) to 2500 nt of sequence upstream of exon 0, a strong putative promoter region is predicted (see Figure 1B). It includes consensus binding sites for NF-KB, SP1 and CAC-BP, as well as a TATA box sequence. According to this prediction the transcriptional start site is located 469 nt upstream of the translational start codon of exon 0.

The sequence conservation between mouse and human, its presence in mouse and human transcribed sequences and the existence of a separate ADAR2 exon 0 in both the mouse and human genome with conserved 5′-splicing consensus confirms that this ADAR2 splice variant probably subserves a conserved function in mammals.

### Exon 0 is strongly conserved in vertebrates and encodes a protein sequence closely related to a functional domain in ADAR3

Interestingly, the exon 0 encoded protein sequence unique to the new alternative splice form of ADAR2 harbors a stretch of positively charged residues that are highly similar in sequence, length and relative position to the so-called R-domain of ADAR3 [12] (see Figure 2). The R-domain of ADAR3 was recently shown to have specific binding affinity for single-stranded RNA [21].

The human and mouse ADAR2 cDNA sequences of the exon 0 encoded R-domain differ in only one nucleotide position and they are 100% identical on the protein level (see Figure 2). The translational Kozak-sequence CATGG is also conserved between human/mouse ADAR2 exon 0 and ADAR3. In addition, the computer program “exoniphy” [23] identifies a highly conserved exon sequence in the ADAR2 gene that coincides with the coding region of that R-domain in exon 0 in human, rat, mouse, dog, orangutan and horse. The exoniphy program identifies evolutionarily conserved protein-coding exons in a multiple alignment using a phylogenetic hidden Markov model, a statistical model that simultaneously describes exon structure and exon evolution.

**Figure 3:**
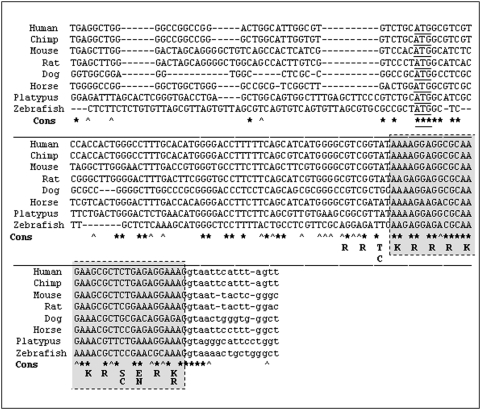
The R-domain and exon 0 are conserved across vertebrates. Vertebrate exon 0 sequence alignment including human, chimp, mouse, rat, dog, horse, platypus and zebrafish. The consensus track indicates residues conserved among all species with an asterisk (*) and those conserved in all higher vertebrates with a circumflex accent (∧). The nucleotide sequence of the R-domain including the translation is shaded; the translational start codon in underlined. The genomic locations of the aligned sequences are: UCSC version hg18, March 2006, chr21:45,396,913–45,397,066 (Homo sapiens); UCSC version panTro2, March 2006, chr21:44,799,898–44,800,051 (Pan troglodytes); UCSC version mm8, February 2006, chr10:76,783,722–76,783,879 (Mus musculus); UCSC version rn4, November 2004, chr20:11,691,863–11,692,020 (Rattus norvegicus); UCSC version canFam2, May 2005, chr31:41,072,028–41,072,166 (Canis familiaris); UCSC version equCab1, February 2007, chr26:1,562,280–1,562,433 (Equus caballus); UCSC version ornAna1, March 2007, Ultra489:519,316–519,487 (Ornithorhychus anatinus); and NCBI assembly Zv7, July 2008, Chromosome 22, NW_001878325.1 (Danio rerio).

Through further database analysis, we are also able to locate a highly similar sequence in the zebrafish genome (Danio rerio; chromosome 22), which maps within the zebrafish ADAR2 gene and also encodes an R-domain protein sequence (see Figure 3). The 5′-splice consensus site is also conserved at the same position as in human, mouse, rat, and other higher vertebrates.

Interestingly, the zebrafish exon 0 sequence displays a 10 bp deletion just downstream of the ATG that aligns with the predicted translational start codon in the mammalian sequences. Further downstream, a second ATG is positioned in frame with the following R-domain sequence and could represent the initiation codon. Alternatively, translation could initiate at the first ATG and lead to premature termination precluding the translation of the R-domain. Furthermore, we cannot formally rule out that the 10 bp deletion seen in the available zebrafish sequence is subject to polymorphisms and another allele that lacks the deletion exists as well. The different configuration of ADAR2 exon 0 in zebrafish could be an indication of evolutionary changes in the regulation of A-to-I RNA editing that took place during the development of higher vertebrates.

The ADAR3 genome architecture is strongly conserved to the one of ADAR2 with respect to the splice donor site directly following the R-domain sequence.

We will refer to this ADAR2 isoform as ADAR2R from here on based on the presence of the highly conserved R-domain.

Another interesting observation is that the sequence of exon 1 that serves as 5′-untranslated region (5′-UTR) in the major splice form of ADAR2, is more strongly conserved across species than other 5′-UTR sequences. Indeed, in ADAR2R, this part of exon 1 is translated and therefore contributes to the ADAR2R protein sequence. This could explain why the sequence is conserved more strongly and further supports the notion that ADAR2 fulfills a conserved function in vertebrates.

### Differential expression of ADAR2R mRNA in human tissues

We next addressed the question where and to what extent ADAR2R is expressed in human tissues. We initially detected the ADAR2R cDNA in mouse brain. Upon retrieving the orthologous human sequence from the databases as described above, we could indeed amplify by RT-PCR the splice-variant specific cDNA from human brain using human ADAR2 specific primers.

**Figure 4:**
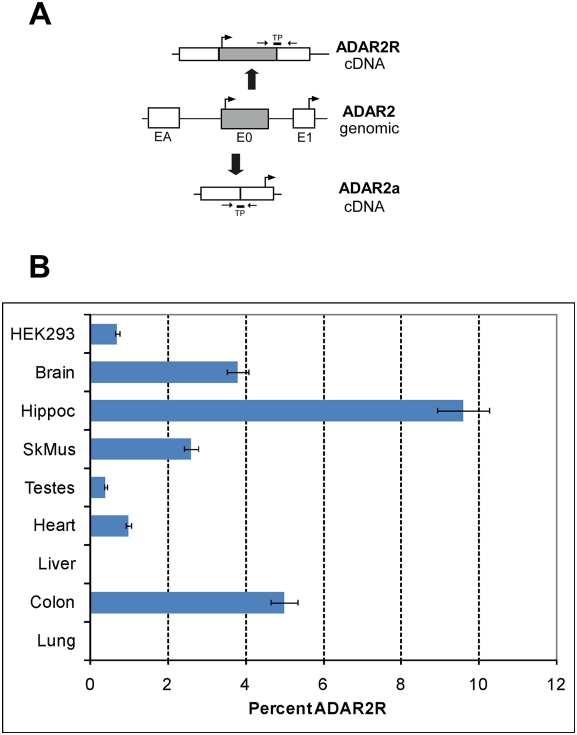
Expression of ADAR2R in human tissues. A) Design of real-time PCR assay for ADAR2 alternative splice forms. Locations of primers and TaqMan probe (TP) are indicated. B) Graphic representation of the ratio of exon 0-encoding mRNA transcripts relative to the total amount of ADAR2 mRNAs in various human tissues by quantitative real-time PCR. The percent values are derived from triplicate assays for three different cDNA concentrations from each tissue.

Subsequently we analyzed through quantitative real time PCR the relative expression of the ADAR2R splice variant compared to all ADAR2 splice variants lacking exon 0 using several human tissue total RNAs as starting material. Figure 4A depicts the real-time PCR strategy where splice-variant specific amplicons are generated using exon specific primers and are then quantified using variant-specific TaqMan probes (ABI). Figure 4B shows the percentage of R-domain encoding mRNAs relative to the total amount of endogenously expressed ADAR2. In liver and lung no detectable signal with ADAR2R specific primers is obtained, whereas the highest levels of the transcript containing exon 0 are detected in hippocampus [9.6±1%] and colon [5.0±0.5%].

These data document that ADAR2R is expressed in various human tissues and that the relative amounts of the R-domain encoding ADAR2 mRNAs differ between cell types. This might be due to the existence of a separate promoter driving the expression of ADAR2R messages or might reflect a change in alternative splicing of ADAR2 pre-mRNAs (see Figure 4A).

The ADAR1 gene is also expressed through alternative promoters of which one is interferon induced. With respect to ADAR2R expression, the currently available evidence does not suggest that this protein isoform is stimulated by interferon. Since the ADAR2R isoform is identical to the ADAR2 major isoform except for the small N-terminal extension, an increase or decrease of ADAR2R upon interferon stimulation of cells would generally result in the up- or downregulation of the overall amount of ADAR2. However, such an effect was not observed in several studies analyzing ADAR expression during interferon induction (see references [18], [19], [28]). ADAR2 expression remains unaltered by interferon action, whereas ADAR1 is strongly induced by interferon.

Due to the small difference in molecular weight between the ADAR2 major protein isoform, several ADAR2 splice variants, and the ADAR2R isoform, the unambiguous detection of endogenously expressed ADAR2R would require an isoform specific antibody. However, such an antibody is not available. In fact, due to the sequence nature of the R-domain (highly repetitive as well as highly conserved between ADAR2R and ADAR3), this strategy may not be successful. When using an antibody that recognizes all ADAR2 splice variants however, a signal that likely represents ADAR2R is detectable above background in brain tissues that show ADAR2R expression as separate band or showing a likely double band (such as figure 3 in Feng et al.[29] and figure 3 in Singh et al. [30]). However, due to the potential presence of other ADAR2 splice variants with similar molecular weights, the use of a general ADAR2 antibody cannot formally prove the presence of ADAR2R. In the future, highly sensitive proteomics mass-spec technology may resolve this issue.

When recombinantly expressing ADAR2R in HeLa and HEK293 cells, we did not detect any differences in general adenosine deaminase activity displayed by ADAR2R compared to the ADAR2a major splice variant (data not shown). If the R-domain in ADAR2R conveys a selective binding affinity to a specific nucleic acid substrate, then a functional difference between ADAR2R and ADAR2a will likely be limited to the activity on that specific target.

### Recoding SNP within R-domain of human ADAR2R

When comparing the cDNA of the initially cloned mouse ADAR2R sequence, we noticed an A-to-G discrepancy changing a genomically encoded Arginine codon (AGG) to a Glycin codon (GGG). Since in mouse this position has not been mapped as a genomic single nucleotide polymorphism (SNP) and the sequence alteration is within the highly conserved R-domain sequence motif, this base discrepancy may represent a site of RNA editing. The ADAR2 pre-mRNA is already known to be subject to selfediting by the ADAR2 protein at another site, where the base modification creates an alternative splice site in intron 1 that leads to the expression of a truncated, nonfunctional protein.

We also noted that within the human ADAR2 exon 0 sequence, there is a recoding SNP annotated that alters an Isoleucin (ATC) to a Valin (GTC) codon upstream of the R-domain. Since this SNP is also of the A-to-G type, there could be A-to-I RNA editing being responsible for some of the observed discrepancies, even though individuals with both alleles of the gene would be able to produce both isoforms without the need for editing.

To test if these two nucleotide positions within the ADAR2 exon 0 sequence may be subject to an RNA-based modification, we performed RT-PCR on human brain total RNA amplifying the exon 0 sequence encompassing both putative editing sites. In parallel, the corresponding genomic region was amplified from genomic DNA prepared from the same specimen that gave rise to the total RNA. This ensures that any SNP can be distinguished from post-transcriptional base modifications.

For both sites, we did not detect any mixed sequence populations when analyzing the gene-specific amplicons (data not shown). This means that at least in human total brain, there is no detectable RNA editing involving these two positions. We cannot formally rule out that RNA editing may occur at low levels (below the detection limit of the RT-PCR sequencing analysis) or selectively within specific cell types, or at specific time points.

## Materials and Methods

### 5′ RACE

For confirmation of the mouse Adar2 cDNA sequence at the 5′-end, a rapid amplification of cDNA ends (RACE) experiment was performed with total RNA isolated from mouse brain using TRIzol-reagent (Invitrogen) according to the manufacturer's protocol. Reverse transcription reactions were performed using Superscript reverse transcriptase (Invitrogen) and mouse Adar2 specific antisense primer R2N2U (5′-GAGACGGATCCCGTTTGATTTCGTTCAGC-3′) located within exon 2 at 45°C for 1 h. The resulting cDNA products were 3′ tailed with oligo(dA) using Terminal Deoxyribonucleotidyltransferase (Boehringer Mannheim). Primers for the subsequent PCR were PCRdT18 (5′-GACACGGTACCACACAACGGT_18_-3′) and R2G12 (5′-CGTCTAGAATATCAGTGCTGCTGGAAC-3′), and 5′-specific PCR amplicons were analyzed for their sequence.

The nucleotide sequences of the human and mouse ADAR2R partial cDNA sequences containing exon 0 were submitted to GenBank (Accession numbers: FJ169505 and FJ169506)).

### Sequence analysis and alignments

The genomic ADAR2 human and mouse sequences were analyzed using the UCSC genome browser [22]. Highly conserved exons were identified within the ADAR2 gene using the exoniphy track within the genome browser [23], which uses a phylogenetic hidden Markov Model that statistically analyses exon structure and exon evolution within multiple alignments [23].

The conservation of exon 0 nucleotide sequences across vertebrate genomes was analyzed using the conservation track of the genome browser, which is based on the phastCons program designed to identify conserved elements in multiply aligned sequences [24]. The zebrafish (Danio rerio) ADAR2 gene sequence corresponding to exon 0 was identified using tblastn (NCBI).

Prediction of promoter regions within the human ADAR2 sequence was performed using the algorithm ProScan, which identifies putative promoters through the localization of transcription factor binding sites [25].

### Quantitative real time PCR

Real-time PCR (TaqMan analysis) was performed on cDNA from human tissues and human HEK 293 cells according to the manufacturer's instructions (Applied Biosystem, USA) and the reaction conditions involved denaturation for 10 min at 95°C, and 45 cycles of amplification with 15 sec at 95°C and 1 min at 60°C. Primers and probes for TaqMan™ quantitative real-time polymerase chain reaction (qRT-PCR) assays, specific for each human ADAR2 splice variant, were designed with Primer Express v1.2 (Applied Biosystems).

Amplification values were determined in triplicates using an ABI prism 7000 (Applied Biosystem). Standard curves were run for all assays to ensure consistent amplification efficacy. The ADAR2R-specific signals were normalised to the ADAR2a assay using the comparative C_T_-method (User Bulletin #2, December 11, 1997 (updated 10/2001); ABI PRISM 7700 Sequence Detection System) and presented as relative expression levels. Primers for ADAR2R splice variant: A2E0F: 5′-GGTATAAAAGGAGGCGCAAGAAG-3′, A2E0R: 5′-GTTTCTTGACTGGCGGAGACT-3′, A2E0M1 (FAM labeled): 5′-CTGAGAGGAAAGACAGAAAC-3′. Primers for the ADAR2 major splice form: A2MF: 5′-CTATTCCCAGTGAGGGTCTTCAG-3′, A2MR: 5′-GGACCAGGCGTGAGACA-3′, A2MM1(FAM labeled): 5′-CATTTACCGCAGGTTTTAG-3′.

### RNA editing analysis

Commercially available matched pairs of total RNA and genomic DNA derived from one individual (Clontech) were analyzed for evidence of RNA editing using standard procedures as described previously [26]. RNA editing analysis was done by direct sequencing of gene-specific, gel-purified RT-PCR products as described [26].

Transient co-expression of human ADAR2a and ADAR2R separately with a minigene for the glutamate receptor subunit GluR-2 R/G editing site [27] in human embryonic kidney cells (HEK293) and HeLa cells was performed as described [27]. Analysis of RNA editing at the R/G site of ectopically expressed GluR-2 transcripts was determined with RT-PCR using GluR-2 specific primers as described above [26].
